# Quality Assurance of Undergraduate Medical Education in Israel by Continuous Monitoring and Prioritization of the Accreditation Standards

**DOI:** 10.5041/RMMJ.10480

**Published:** 2022-07-31

**Authors:** Jochanan Benbassat, Reuben Baumal, Robert Cohen

**Affiliations:** 1 Department of Medicine (retired), Hadassah—Hebrew University Medical Centre, Jerusalem, Israel; 2 Department of Laboratory Medicine and Pathobiology (retired), University of Toronto, Toronto, Ontario, Canada; 3 Center of Medical Education (retired), Hebrew University—Hadassah Faculty of Medicine, Jerusalem, Israel

**Keywords:** Accreditation of medical schools, Israel, medical education, quality assurance

## Abstract

External accreditation reviews of undergraduate medical curricula play an important role in their quality assurance. However, these reviews occur only at 4–10-year intervals and are not optimal for the immediate identification of problems related to teaching. Therefore, the Standards of Medical Education in Israel require medical schools to engage in continuous, ongoing monitoring of their teaching programs for compliance with accreditation standards. In this paper, we propose the following: (1) this monitoring be assigned to independent medical education units (MEUs), rather than to an infrastructure of the dean’s office, and such MEUs to be part of the school governance and draw their authority from university institutions; and (2) the differences in the importance of the accreditation standards be addressed by discerning between the “most important” standards that have been shown to improve student well-being and/or patient health outcomes; “important” standards associated with student learning and/or performance; “possibly important” standards with face validity or conflicting evidence for validity; and “least important” standards that may lead to undesirable consequences. According to this proposal, MEUs will evolve into entities dedicated to ongoing monitoring of the education program for compliance with accreditation standards, with an authority to implement interventions. Hopefully, this will provide MEUs and faculty with the common purpose of meeting accreditation requirements, and an agreed-upon prioritization of accreditation standards will improve their communication and recommendations to faculty.

## INTRODUCTION

To assure and improve the quality of their undergraduate programs, medical schools have established departments/offices for education (referred to as medical education units, MEUs),^1^ and most countries conduct periodic external reviews to ascertain that medical schools meet predetermined accreditation standards.^2^–^5^ In Israel, the Council for Higher Education is the official national authority on issues related to higher education. It evaluates the quality of teaching at the various institutions, including the six medical schools, using the standards of medical education in Israel (SMEI).^5^

The function, structure, and staffing of MEUs vary among medical schools in the West. In addition to quality assurance, MEUs are expected to contribute to other aspects of education, such as integration of new methods of instruction and their evaluation, enhancing scholarly activity, creating and monitoring the institution’s vision and mission, and recommending reforms and innovations. Some MEUs are subdivisions of the office of the Dean; others are independent academic departments; and still others, such as in Sydney, Australia,^6^ have evolved into centers or schools of public health. In 2008, North American MEUs employed on average five professional and faculty staff who were supported by university funds, research and training grants, and contracts with other institutions.^7^

The external reviews for accreditation require medical schools to perform a self-evaluation of their programs. This self-evaluation helps the accreditation committee prepare for site visits that include reviews of documentation, inspections, and meetings with faculty and students. After visits, the committee provides the deans with its initial findings, and, several months later, with final recommendations. Although its main purpose is to improve the educational processes in medical schools, accreditation is subject to several types of uncertainties and criticism.

Firstly, accreditation visits in North America occur at 4–10-year intervals and do not identify problems promptly as they occur.^8^

Secondly, the accreditation standards are not equally important. Experts reportedly agreed that only 14 of the 150 standards of the World Federation of Medical Education (WFME) were essential, and disagreed regarding the importance of the remaining standards.^9^ The UK General Medical Council (GMC) and WFME distinguish between standards that “must” and those that “should” be met. The Liaison Committee of Medical Education (LCME) discerns between standards that, if not complied with, place a teaching program at “immediate” and “lesser” risks. Nevertheless, we know of no agreed-upon taxonomy of standards, and consequently the differences in their importance do not figure meaningfully in the accreditation process.

Thirdly, a 2021 review of the literature of the impact of accreditation on medical teachers indicated that even though faculty and students recognized the merits of accreditation (e.g. switching to active learning), they also recognized its unintended negative consequences (e.g. faculty distraction from teaching in favor of accreditation bureaucracy). Faculty and students thought that a dedicated unit overseeing the quality assurance and preparation for accreditation would improve the management of the curriculum.^10^

Finally, accreditation and re-accreditation have been implemented in North America for 80 years. However, in Israel, a single accreditation review was conducted in 2007 of the then four medical schools, and, only recently, the two newly founded medical schools had their first accreditation visit along with the older schools of medicine. There is no established tradition for the implementation of the SMEI,^5^ for self-study of the curriculum, or for discerning between important and less important standards.

In this paper, we propose that MEUs are assigned the task of overseeing the preparation for accreditation by continuous self-evaluation/monitoring of the implementation of the teaching programs in the medical school. Indeed, such continuous monitoring has already been shown in ten United States medical schools to improve the learning environment, career advising, teaching the physical examination, clerkship feedback, and communication with faculty and other stakeholders.^11^ Furthermore, we suggest a four-level classification of standards according to the strength of evidence for their importance, derived from published review articles. Although the review of the literature was only preliminary, we hope that our suggestions will open a discussion of the function, structure, staffing, funding, and expectations from MEUs, and of the relative importance and need for prioritization of the accreditation standards.

## MEDICAL EDUCATION UNITS IN ISRAEL

In 2016, MEUs in Israel were either independent departments, units of the office of the Dean, or combinations thereof, and they varied in the number of full-time and part-time academic (MD and PhD) staff.^12^ Beyond other activities, Israeli MEUs conducted workshops for faculty development and were involved in the teaching of the behavioral sciences and clinical skills. In addition, there were independently staffed units of two or more full-time faculty/professionals who reported to the office of the Dean and advised on student assessment, implemented multiple-choice tests, provided faculty with feedback based on students’ rating of teaching, and offered multimedia, simulations, and support in computer use.^12^

As of January 2022, all six of Israel’s medical schools monitored the quality of teaching based on student ratings of instruction; additionally, in three schools, student debriefing, focus groups, and faculty reports were also used. Medical schools did not implement a continuous review of compliance with accreditation standards, as proposed in 2015 by Barzansky et al.,^8^ and as required by the SMEI (standard 1.1) to engage “in ongoing … continuous quality improvement processes … [and] ensure effective monitoring of the medical education program’s com-pliance with accreditation standards.”^5^(p Barzansky et al.^8^ raised the question of whether this monitor-ing should be guided by all or only selected accred-itation standards and, if the latter, how these should be chosen. In the following sections, we attempt to answer this question by proposing a prioritization of the standards of accreditation based on the strength of evidence for their importance.

## PROPOSED PRIORITIZATION OF ACCREDITATION STANDARDS BY STRENGTH OF EVIDENCE FOR THEIR Validation

A straightforward validation of the accreditation standards would demonstrate their association with student well-being and patient health outcomes. However, until 2000, most measures of teaching addressed only their face validity and their association with student learning and satisfaction, and only 0.7% of the studies assessed patient outcomes.^13^ Only in the last two decades did research use patient health outcomes for validation of teaching programs, and the advent of electronic medical records offers potential use of big data to improve care by linking clinical outcomes to educational programs.

We propose a four-tier prioritization of the SMEI^5^ according to the level of their validation in the literature (Table 1). Level 1 contains the “most important” standards shown to be associated with student well-being and, in practicing doctors, with improved patient health outcomes. Level 2 contains “important” standards associated with student learning and/or performance. Level 3 consists of “possibly important” standards with face validity or conflicting evidence for validity, and level 4 comprises the “least important” standards, which are subject to controversy and may lead to unintended adverse consequences.

**Table 1:** Proposed Classification of the Standards for Medical School Accreditation by Strength of Validation.

Accreditation Standard (SMEI Standard #)^5^	Justification for Inclusion into the Level of Importance
***Level 1. Most important accreditation standards:*** ***Standards associated with student well-being or patient health outcomes***	
A medical school ensures that its medical education program occurs in professional, respectful, and intellectually stimulating academic and clinical environments (3.1–3.5)	A positive perception of the learning environment is associated with students’ reduced burnout and improved quality of life, resilience, preparedness for practice, and well-being^14^–^21^

Instruction and assessment of students’ communication skills (7.8)	Teaching communication skills improves patients’ satisfaction with care, adherence to recommendations, and health outcomes in hypertensive patients^22^–^24^

Use of simulation equipment and facilities (5.5)	Simulation in training is superior to traditional training; the use of skill simulation laboratories leads to small-to-moderate improvements in patient benefits^25^–^27^

Assessment of student achievement employs a variety of measures of knowledge, competence, and performance (9.1–9.7)	Success in examinations is associated with improved performance on USMLE, internship, residency, clinical practice, and patient outcomes^28^–^33^

An effective system of personal counseling for medical students (11.5)	Student well-being initiatives aimed at improving the learning environment, and teaching how to use psychological and emotional support resources reduce student depression and anxiety rates^34^

***Level 2: Important accreditation standards:*** ***Standards associated with student learning and/or performance***	

Methods of pedagogy (8.4):	An association has been reported between these teaching methods and various aspects of learning
Online lectures	35
Self-directed learning (6.4)	36
Evidence-based medicine	37,38
Problem-based learning	39–43
Social determinants of health (6.1, 7.7)	44
Decision-support systems	45

Formative examinations with feedback (9.7)	Formative examinations improve clinical performance, learning outcomes, and development of professional behavior^46^,^47^

Continuing professional development programs for faculty (4.1–4.4)	Faculty development programs affect faculty learning and change of behavior^48^

Faculty receives feedback on teaching (4.4)	Use of student feedback to course directors improves teaching programs^49^–^52^

Instruction in patient care is provided in ambulatory and hospital settings (6.5)	Students rate clerkships in a single general practice setting higher than the traditional clerkships with respect to teaching, feedback, role-modeling, and patient-centered experiences^53^

Strategic planning and continuous quality improvement (1.1)	Monitoring for compliance with accreditation standards improves the learning environment, career advising, teaching history and physical examination, and clerkship feedbacks^11^

***Level 3: Possibly important accreditation standards:*** ***Standards with face validity, or with conflicting evidence for association with student learning***	

A medical school defines its objectives and makes them known to all medical students and faculty (6.1)	Although defining learning objectives has compelling face validity, there is only conflicting evidence for their association with student learning 54–56

Methods of pedagogy (8.4):	These teaching methods are at least as effective as traditional learning in improving the behavior of healthcare professionals
Web-based instruction	57
Flipped classrooms	58
Case-based learning	59
Small-group teaching	60

Quality of examinations (reliability; questions that test higher cognitive levels)	The quality of examinations probably affects student evaluations

A medical school has a sufficient number of faculty in leadership roles and senior administrative staff with the skills, time, and administrative support necessary to achieve the goals of the medical education program (2)	A sufficient number of faculty and administrative support have a compelling face validity

***Level 4: Least important accreditation standards:*** ***Standards with possible unintended consequences***	

Admission policies: Selecting applicants with personal and emotional attributes necessary for them to become competent physicians (10.1–10.5)	There is conflicting evidence that selection for non-cognitive attributes predicts students’ performance. Such selection may reduce the self-esteem of rejected applicants and may not justify the expensive selection procedure^61^–^65^

Use of student ratings of individual teachers to inform academic promotions (4.4)	There is conflicting evidence that student ratings of individual teachers are associated with teaching effectiveness;^49^–^52^,^66^,^67^ the use of student ratings of individual teachers to inform academic promotions may contribute to student–faculty alienation

SMEI, Standards for Medical Education in Israel; USMLE, United States Medical Licensing Examination.

# numbers of the accreditation standards.

### Level 1: Most Important Accreditation Standards

The SMEI require a “professional, respectful, and intellectually stimulating academic and clinical environment” (standard 3) that “allows medical students to report … incidents of harassment or abuse without fear of retaliation” (standard 3.5).^5^(p As early as 1973, Atkinson noted that preceptors of the clerkship rotations varied between those viewing students as subordinates “… [whose] progress towards qualification was … a long obstacle race” and those viewing learners as student-physicians “treated in an egalitarian manner, and … being groomed for full professional status as soon as possible.”^68^(p This impression is supported by the variability in students’ appreciation of their learning environment among different medical schools.^14^,^15^ The SMEI requirement is consistent with evidence that student learning environment assessments are inversely associated with student burnout^14^ and correlate with student learning,^15^,^16^ quality of life, resilience, positive attitudes towards the course, preparedness for practice, and well-being.^17^,^18^ Evidence also suggests that the learning environment, rather than students’ personality traits, is the main source of students’ distress.^19^ As late as 2019, it was reported that student humiliation^20^ and neglect^21^ by faculty were frequent in clinical teaching settings. We believe that it is impossible to ignore students’ distress while teaching them how to be sensitive to patients’ distress and, if medical students are humiliated, it is equally impossible to teach them how to respect patients. *Therefore, we consider the quality of the learning environment and student experiences during the clerkship rotations in terms of their perceived relationship with their preceptors as the most important standard of accreditation*.

The SMEI require “instruction and assessment of students’ communication skills with patients, families, colleagues and other health professionals” (standard 7.8)^5^(p and “… the use of … simulations equipment and facilities” (standard 5.5).^5^(p Patient health outcomes improved when practicing doctors were taught communication skills^22^–^24^ and used simulations during their training.^25^–^27^ Accreditation also requires “that the assessment of student achievement employs a variety of measures of knowledge, competence, and performance, systematically and sequentially applied throughout the medical school” (standard 9.1).^5^(p This requirement is supported by evidence that examination performance in medical school predicts internship performance, the United States Medical Licensing Examination (USMLE), and clinical practice.^28^,^29^ Academic achievements before admission to medical school have also been shown to predict grades on preclinical examinations, assessments during the clerkship rotations, and post-graduate evaluations.^30^,^31^ There is also evidence that patients treated by certified cardiologists^32^ and anesthesiologists^33^ who had passed board examinations have better health outcomes than patients treated by non-certified care providers.

Examinations not only assess students’ knowledge, skills, and attitudes, they also affect learning, because students perceive the content of examinations as reflecting faculty priorities.^69^ Evidence suggests that examinations are more powerful drivers of student learning than instructional format.^70^ Hence the need for a *variety* of measures of competence, such as supervised patient interviews, long case presentations, objective structured clinical examinations, high-fidelity simulations, assessments of students’ professionalism, and the ability for self-directed learning.

The SMEI require “… an effective system of personal counseling for its medical students that includes programs to promote their well-being and to facilitate their adjustment to the physical and emotional demands of medical education” (standard 11.5).^5^(p This requirement is consistent with the report that student well-being initiatives aimed at reducing stressors, upgrading the learning environment, managing stress, and using psychological and emotional support led to an 85% reduction in depression rates and a 75% decrease in anxiety rates in first-year medical students during a 10-year follow-up.^34^

### Level 2: Important Accreditation Standards

The accreditation standards require that “methods of pedagogy utilized for each segment of the curriculum, as well as for the entire curriculum, [be] subjected to periodic evaluation” (standard 8.4).^5^(p There is evidence that using online lectures,^35^ promoting self-directed learning,^36^ teaching evidence-based medicine,^37^,^38^ and teaching decision-support systems^45^ improve learning, knowledge, and attitudes. The COVID-19 pandemic has affected the delivery of medical education with a shift towards online teaching platforms. It has been suggested to incorporate online teaching methods within traditional face-to-face medical education, thereby maximizing the benefits of both, and promoting the shift in medical practice toward virtual consultations.^71^

Problem-based learning (PBL) is one of the most studied methods of pedagogy. A review of the 1972–1992 literature indicated that, when compared with conventional instruction, PBL is more enjoyable and its graduates perform as well on clinical examinations and faculty evaluations; but they score lower on basic sciences examinations, with gaps in the knowledge base that could affect practice outcomes.^39^ More recent studies have similarly indicated that PBL has positive effects on physician competence^40^ and the learning environment.^41^ A 2010 review indicated that 12 of 15 studies found no differences between PBL and traditional learning in knowledge acquisition; however, a few studies found improved clerkship or residency performance.^42^ Finally, a 2019 review indicated that merging traditional lecture-based teaching and PBL led to better student performance and satisfaction than either PBL or traditional teaching alone.^43^

Standard 6.1 requires that “[t]he curriculum provides a broad-base education in … various ethical, cultural, behavioral and socioeconomic subjects pertinent to medicine,”^5^(p and standard 7.7 requires specifying “how students are prepared for their role in addressing the medical consequences of common societal problems, for example, providing instruction in the diagnosis, prevention, appropriate reporting and treatment of violence and abuse. Students are instructed in the social determinants of health.”^5^(p A recent literature review indicated that most reviewed studies concluded that teaching the social determinants of health was effective in terms of student performance or self-reported ability to identify social determinants of health.^44^

Accreditation standards require that each medical student be “assessed and provided with formative feedback early enough to allow sufficient time for remediation” (standard 9.7).^5^ There is undisputed evidence that formative examinations improve clinical performance,^46^ learning,^47^ and professional behavior.^72^ The SMEI also require that “[t]he faculty members of a medical school are qualified through their education, training, experience, and continuing professional development” (standard 4); that the “recruitment and development of a medical school’s faculty takes into account its mission, the diversity of its student body, and the populations that it serves” (standard 4.2); and that “[o]pportunities for professional development are provided to enhance faculty members’ skills and leadership abilities in teaching and research” (standard 4.4).^5^(p A recent review of studies of staff-development programs indicated that participants rated most of these programs highly, and some of them also reported enhanced confidence and comfort with their teaching, higher student ratings, and improved academic ability in terms of publications and conference presentations.^48^

Standard 4.4 states: “Faculty members receive feedback on teaching.”^5^(p Although a subject of controversy, students’ ratings of teaching agree with several credible indicators of teaching effectiveness: student learning, student comments, alumni ratings, and ratings of teaching by outside observers.^49^ Furthermore, students’ ratings have been reported to discern between individual teachers,^50^ and to improve teaching programs,^51^ performance of individual teachers,^49^ and clinical teaching.^52^ On the other hand, students’ ratings may be influenced by factors unrelated to teaching effectiveness, such as course workload,^66^ student motivation for taking the course, and anticipated success in examinations.^67^ However, while students’ feedback on courses, clinical teaching, and individual teachers may lead to improved teaching performance, using students’ ratings of individual instructors to inform and influence academic promotions may have undesirable consequences, as discussed in the last paragraph of the section Level 4: Least Important Standards.

Currently, clinical training is performed through bedside teaching in hospitals and field exercises in the community. Standard 6.5 requires that “[i]nstruction and experience in patient care are provided in both ambulatory and hospital settings.”^5^(p Some medical schools have introduced into their programs “integrated clerkships,” a 6–12-month experience in a single general practice setting. Students are expected to follow their patients through the entire healthcare continuum, including hospital admission, to meet the curriculum requirements on the various medical disciplines. Comparative studies have indicated that students rated a year-long, integrated clerkship higher than the traditional, block clerkships with respect to teaching, feedback, role-modeling, and patient-centered experiences; students of integrated clerkships outperformed those of block clerkships in clinical skills and performed similarly on the USMLE.^53^ To the best of our knowledge, while all medical schools in Israel include primary care clerkship rotations, no medical school has substituted block clerkship rotations with longitudinal integrated clerkships.

### Level 3: Possibly Important Accreditation Standards

Standard 6.1 requires that “[a] medical school defines its objectives and makes them known to all medical students and faculty.”^5^(p The need for pre-determined learning objectives has compelling face validity because intended outcomes underpin all teaching, learning, and assessment activities. However, the association between formal objectives and student outcomes is uncertain. While defining learning objectives has been reported to improve student learning,^54^ another study showed that providing learning objectives did not improve students’ performance in an emergency ward,^55^ and using learning objectives did not enhance ward evaluations, examination success, and student satisfaction.^56^

As stated earlier, standard 8.4 requires “methods of pedagogy utilized for each segment of the curriculum, as well as for the entire curriculum.”^5^(p Evidence suggests that web-based instruction,^57^ flipped classrooms,^58^ case-based learning,^59^ and small-group teaching^60^ are at least as effective as traditional learning in improving healthcare professionals’ behavior.

Finally, the requirement for “… a sufficient number of faculty in leadership roles and of senior administrative staff with the skills, time, and administrative support necessary to achieve the goals of the medical education program” (standard 2)^5^(p has compelling face validity. Even a program with a superb curriculum cannot maintain itself without resources and governance. It makes sense that student services affect learners’ well-being, and efforts to improve the quality of education will affect students’ learning.

### Level 4: Least Important Accreditation Standards

Accreditation standards require medical schools to implement *admission policies* aimed at selecting applicants with academic, personal, and emotional attributes necessary for them to become competent physicians (standards 10.1–10.5).^5^ There is undisputed evidence that students with top academic achievements before admission to medical school outperform other students not only during the first three years in medical school but also during the clerkship rotations.^30^,^31^

However, the different attempts to identify the applicants’ attributes deemed necessary for becoming a competent physician have led to the present wide variability in admission policies. On the one hand, these attempts respond to social expectations. They attest to the mission and values of the medical school, and a 2020 Dutch study found that applicants admitted via a selection procedure for personal attributes outperformed initially rejected lottery-admitted students by 12%–19%.^61^ However, a different study, also from Holland, found that selected students did not outperform lottery-admitted students and questioned the justification of the expensive selection procedure.^62^ Furthermore, a 2016 systematic review of the literature found that the few longitudinal predictive validity studies available lacked sufficient detail regarding the outcome variables,^63^ and it has been argued that a declared quest for personal attributes may affect the self-esteem of rejected applicants, particularly if they are left wondering if indeed there is something wrong with their character.^64^ Finally, society needs not only clinicians but also researchers and a variety of other medical specialists. Different careers require different personal attributes.^65^

We stated earlier that students’ ratings of individual teachers (standard 4.4) may provide useful feedback and improve teaching effectiveness.^49^ However, such feedback may also be biased by workload, student motivation, and anticipated success on examinations. Therefore, while student ratings of courses and student feedback to individual teachers should be considered an important standard, we believe that the use of student ratings to inform decisions for academic promotions may be humiliating and contribute to student–faculty alienation, and should be considered among the least important standards.

## DISCUSSION

Two suggestions emerge from the presented overview. The first one is to assign to MEUs the task of monitoring the implementation of the curriculum. Beyond ascertaining its accord with accreditation standards, MEUs would attend to the relationship between their medical school with the regulatory authorities (Ministry of Health), and professional authorities (Scientific Council).

In 2010, Chassin et al.^73^ proposed four criteria for measuring quality of patient care. These criteria require evidence that the measure, firstly, is associated with improved clinical outcomes; secondly, reveals whether the evidence-based care process was provided; thirdly, addresses a process proximate to the outcome (e.g. appropriately administered medications, rather than appropriate diagnostic tests); and fourthly, has few or no unintended adverse consequences. We suggest applying the first, third, and fourth of these criteria to the SMEI and using the proposed four-level classification of the accreditation standards in the monitoring of teaching programs of Israeli medical schools.

Non-compliance with Level 1, the “most important” standards (associated with student well-being and/or improved patient health outcomes) and Level 2, “important” standards (associated with student learning and/or performance), would require urgent attention, and their correction should take precedence over non-compliance with the remaining standards. For example, earlier in this paper, we referred to our belief that the perceived quality of the clinical learning environment is the most important standard of accreditation. The MEUs can obtain insight into this environment through student debriefing, focus groups, and student surveys aimed at obtaining information on students’ reflections on what they find difficult, their experiences, critical incidents, learner–faculty relationship, and the degree to which faculty support students in distress at all times and especially during clinical rotations. Negative student perceptions of their learning environment would justify immediate remediation.

The proposed prioritization is consistent with the recommendation of “evidence-guided education,”^74^ whereby the choice of learning objectives and teaching content should be derived from patient health outcomes, rather than from tradition and opinion. However, our proposal is only partly consistent with previously identified important accreditation standards.^9^,^75^ Similar to our proposal, the previously identified important accreditation standards were teaching clinical skills and assessment of students’ learning. Unlike our proposal, they did not identify as important students’ perceptions of their learning environment.

Continuous monitoring of teaching program implementation would assure the outcomes of external evaluations by accreditation and re-accreditation committees. However, even when faculty understand the importance of meeting these standards, criticism is likely to generate confrontations. We have repeatedly heard faculty blame MEU members for being oblivious of the realities of clinical practice, and MEU members claim that clinicians are ignorant of the basic principles of teaching. By defining one of the MEU functions as continuous monitoring of the curriculum and the degree of its accord with accreditation standards, this polarization may be reduced since both MEU and faculty members would be united in a common purpose, to wit, meeting accreditation standards.

## LOOKING BEYOND ISRAEL

Discussions of the function of MEUs, and of the relative importance of the teaching standards, are germane also for countries with a longer tradition of accreditation reviews than Israel. Hopefully, such discussions will lead to an agreement regarding MEUs’ authority to implement the accreditation standards, and rapport between MEUs and the office of the Dean.

Monitoring of the curriculum is of no value without a mechanism in the medical school and university hierarchy of ensuring that the elicited information is promptly acted upon. Hence we propose the creation of MEUs, with appropriately trained staff and budget, the foremost function of which would be the continuous evaluation of the implementation of the teaching program, and helping faculty correct detected flaws. The MEUs would be part of the governance of the medical school and have the authority to implement interventions.

However, we have no certain answer to the question from whom MEUs would draw their authority. The term “self-evaluation” implies that they would have the backing and support of the Dean. However, in Israel, deans are elected for short terms, and most of them have limited knowledge of how to assess teaching programs. To be effective, MEUs cannot risk being vetoed by the Dean, particularly when she or he cancels a specific effort to improve the educational process. Therefore, policies need to be developed that would establish a meaningful role for MEUs in medical schools. For example, MEUs may draw authority from university institutions that would have to rule in cases of disagreement between the MEU and the Dean. Hopefully, such cases would be rare and exceptional; however, we feel that MEUs, although part of the school governance, should not be subordinate to the office of the Dean.

Certainly, the proposed taxonomy of accreditation standards will generate criticism and various degrees of disagreement. However, we believe that some type of categorization of the accreditation standards is needed to discern between their importance and to identify standards that may lead to undesirable consequences. Specifically, future research should explore the following three areas of uncertainty: firstly, how the current block clerkship rotations compare with integrated clerkships in providing students with clinical training and with exposure to patients with common disorders; secondly, whether the quest for non-academic attributes in medical school applicants justifies its cost; and finally, how to assess the contribution of individual faculty members to the implementation of the undergraduate teaching program.

## Abbreviations

GMC: General Medical Council
LCME: Liaison Committee of Medical Education
SMEI: Standards of Medical Education in Israel
MEUs: medical education units
PBL: problem-based learning
USMLE: United States Medical Licensing Examination
WFME: World Federation of Medical Education.
